# Breeding of Ca_v_2.3 deficient mice reveals Mendelian inheritance in contrast to complex inheritance in Ca_v_3.2 null mutant breeding

**DOI:** 10.1038/s41598-021-93391-6

**Published:** 2021-07-07

**Authors:** Anna Papazoglou, Christina Henseler, Karl Broich, Johanna Daubner, Marco Weiergräber

**Affiliations:** 1 Experimental Neuropsychopharmacology, Federal Institute for Drugs and Medical Devices (Bundesinstitut für Arzneimittel und Medizinprodukte, BfArM), Kurt-Georg-Kiesinger-Allee 3, 53175 Bonn, Germany; 2 Federal Institute for Drugs and Medical Devices (Bundesinstitut für Arzneimittel und Medizinprodukte, BfArM), Kurt-Georg-Kiesinger-Allee 3, 53175 Bonn, Germany

**Keywords:** Genetics, Physiology, Molecular medicine

## Abstract

High voltage-activated Ca_v_2.3 R-type Ca^2+^ channels and low voltage-activated Ca_v_3.2 T-type Ca^2+^ channels were reported to be involved in numerous physiological and pathophysiological processes. Many of these findings are based on studies in Ca_v_2.3 and Ca_v_3.2 deficient mice. Recently, it has been proposed that inbreeding of Ca_v_2.3 and Ca_v_3.2 deficient mice exhibits significant deviation from Mendelian inheritance and might be an indication for potential prenatal lethality in these lines. In our study, we analyzed 926 offspring from Ca_v_3.2 breedings and 1142 offspring from Ca_v_2.3 breedings. Our results demonstrate that breeding of Ca_v_2.3 deficient mice shows typical Mendelian inheritance and that there is no indication of prenatal lethality. In contrast, Ca_v_3.2 breeding exhibits a complex inheritance pattern. It might be speculated that the differences in inheritance, particularly for Ca_v_2.3 breeding, are related to other factors, such as genetic specificities of the mutant lines, compensatory mechanisms and altered sperm activity.

## Introduction

Voltage-gated Ca^2+^ channels (VGCCs) play an essential role in various physiological and pathophysiological processes, such as excitation–contraction coupling, excitation-secretion coupling, neurotransmitter release, regulation of gene expression, developmental processes and reproduction^1–6^. The fine tuning of intracellular/cytosolic Ca^2+^ concentrations is a prerequisite for triggering specific subcellular, cellular and supracellular responses in a complex spatiotemporal manner^1,4,7,8^. The distinct electrophysiological characteristics of VGCCs together with their complex spatiotemporal distribution guarantee this fine tuning of Ca^2+^ entry in various cell types of the organism and mediate their broad spectrum of functional implications^1,2,4,6^. Ten different pore-forming Ca_v_-α_1_ subunits have been cloned and they have been subdivided due to their activation threshold into seven high voltage-activated (HVA) and three low voltage-activated (LVA) channels. The HVA channels are further segregated into *long-lasting* (L-Type) Ca_v_1.1-Ca_v_1.4 VGCCs and Non-L-type Ca_v_2.1-Ca_v_2.3 channels ^1,4^. The LVA group consists of Ca_v_3.1-Ca_v_3.2 channels. In the cellular context, the pore-forming Ca_v_-α_1_ subunits are often associated with various auxiliary subunits, such as α_2_δ, β and γ, building up a VGCC complex. The auxiliary subunits are capable of modulating the pharmacological and electrophysiological properties of the underlying pore-forming Ca_v_-α_1_ subunit^9,10^. Further structural and functional modifications originate from alternative splicing processes and post-translational modifications, such as protein cleavage or interconversion phenomena due to phosphorylation/dephosphorylation^11–13^. In order to get more detailed insight into the physiological relevance of the various VGCCs, scientific groups around the world have inactivated the different Ca_v_-α_1_ subunits. These studies have tremendously increased our understanding on the role of VGCCs and their involvement in the etiopathogenesis of animal and human diseases^1,5,14^.

Mouse lines lacking the Ca_v_2.3 or the Ca_v_3.2 VGCCs have first been generated and described 17–20 years ago and many physiological/pathophysiological implications of both channels were characterized in these models. Ca_v_2.3 knockout mice, for example, exhibit a complex phenotype including, i.a., impaired pancreatic beta cell function and glucose tolerance^15–17^, cardiac arrhythmia and altered autonomic regulation^18–20^, reduced seizure susceptibility^21–27^, dysregulation in hippocampal theta genesis and altered theta architecture^28,29^, impaired presynaptic long‐term potentiation (LTP)^30^, distorted circadian rhythmicity and sleep^31,32^, altered myelinogenesis^33^, modified (neuropathic) pain perception^34–36^, enhanced fear^37^ and altered auditory information processing^38,39^. Ca_v_2.3 VGCCs also serve as key factors in regulating neuronal firing in the CNS, i.e., the tonic, intermediate and burst firing modes and modulate facultative neuronal oscillatory activity in specific neuronal ensembles and networks^40–45^.

The phenotype of Ca_v_3.2 deficient mice is characterized, i.a., by alteration of mechanoreception^46^ and pain response^47–50^, age-induced endothelial dysfunction^51^, retinal dysfunction^52^, (sensory) neuronal hyperexcitability^53–55^, elevated anxiety, impaired memory and reduced sensitivity to psychostimulants^56^. Ca_v_3.2 was also reported to be involved in epileptogenesis/ictogenesis^57–59^. In addition, longitudinal body weight analysis indicated a complex developmental impairment, particularly in Ca_v_3.2^−/−^ mice, which could be related to the cardiovascular phenotype. The latter includes coronary arteriole constriction and focal myocardial fibrosis^60,61^. Recently, we also demonstrated that Ca_v_3.2 deficient mice exhibit altered auditory information processing^62,63^ and alterations in theta genesis and theta architecture^64,65^.

It has recently been reported by Alpdogan et al. (2020)^66^ that inbreeding of both Ca_v_2.3 and Ca_v_3.2 deficient mice exhibits non-Mendelian inheritance, e.g., for Ca_v_3.2^+/−^ × Ca_v_3.2^+/−^ and for Ca_v_3.2^+/−^ × Ca_v_3.2^−/−^ offspring with significant reduction of Ca_v_3.2^−/−^ animals. For Ca_v_2.3^+/−^ × Ca_v_2.3^+/−^ breeding, Alpdogan et al. (2020)^66^ reported a deviation from Mendelian inheritance for heterozygous (HT) male mice, but not for Ca_v_2.3^−/−^ animals. We have been using the same Ca_v_3.2 null mutant line as Alpdogan et al. (2020)^66^ and an alternative Ca_v_2.3 null mutant line for several years with a total number of 926 and 1142 offspring, respectively. Based on our Ca_v_2.3 and Ca_v_3.2 breedings and genotyping results, we analyzed our data for potential deviations from Mendelian inheritance in both lines.

## Results

### Ca_v_3.2 breeding results and characteristics of inheritance

**Figure 1:**
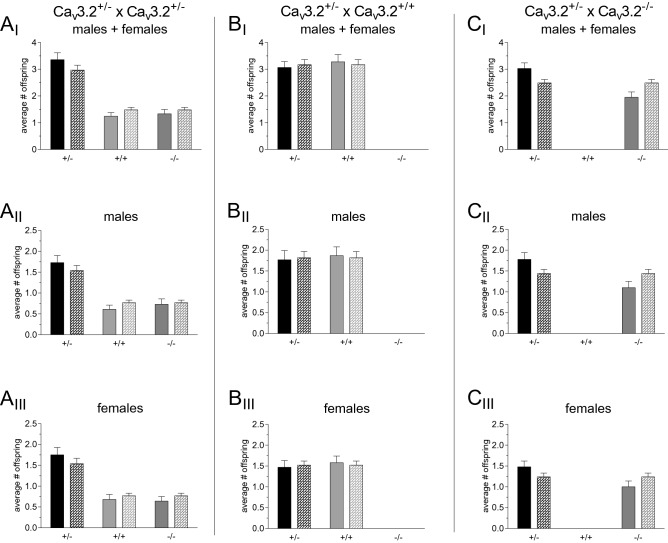
Real and theoretical average number of offspring from different Ca_v_3.2 breeding schemes. **(A**_**I**_**–C**_**I**_) Breeding results for both sexes using a Ca_v_3.2^+/−^ × Ca_v_3.2^+/−^, Ca_v_3.2^+/−^ × Ca_v_3.2^+/+^ and Ca_v_3.2^+/−^ × Ca_v_3.2^−/−^ breeding scheme. In addition, these data were analyzed separately for male offspring (**A**_**II**_**–C**_**II**_) and female offspring (**A**_**III**_**–C**_**III**_). Plain bars indicate real average offspring number, patterned bars indicate the theoretical average offspring number based on the Mendelian inheritance.

Ca_v_3.2 mutant mice were bred for eight years in different projects of our group (see^62–64,67^). Ca_v_3.2^+/+^, Ca_v_3.2^+/−^ and Ca_v_3.2^−/−^ mice were generated using three different breeding schemes, i.e., Ca_v_3.2^+/−^ × Ca_v_3.2^+/−^, Ca_v_3.2^+/−^ × Ca_v_3.2^+/+^, and Ca_v_3.2^+/−^ × Ca_v_3.2^−/−^. In total, 926 offspring from 164 litters were analyzed. For the Ca_v_3.2^+/−^ × Ca_v_3.2^+/−^ breeding scheme (including both sexes) with 344 offspring from 58 litters, a deviation from Mendelian inheritance was detected with an increase of Ca_v_3.2^+/−^, and a decrease of Ca_v_3.2^+/+^ and Ca_v_3.2^−/−^ mice compared to the Mendelian distribution (Fig. 1A_I_, Table 1A, Suppl. Tab. 1A). Interestingly, a sex-specific analysis of the related breeding results did not confirm this non-Mendelian inheritance in both combined sexes (Fig. 1A_II_ for ♂, Fig. 1A_III_ for ♀, Table 1, Suppl. Tab. 2 and 3).

**Table 1:** Results of Chi-square testing for Ca_v_3.2 and Ca_v_2.3 breeding. Breeding results for Ca_v_3.2 (see Suppl. Tab. 1–3) and Ca_v_2.3 (see Suppl. Tab. 4–6) were analyzed using the Chi-square test to check for Mendelian inheritance. For details on the statistical procedure see Montoliu et al. (2012)^115^ Power values are given in brackets.

(A) Ca_v_3.2 Breeding scheme	♂ + ♀	♀	♂
Ca_v_3.2^+/−^ × Ca_v_3.2^+/−^	*p* *=* *0.043* *(non-Mendelian)* *[0.6062]*	**p** **=** **0.183** **(Mendelian)** **[0.3591]**	**p** **=** **0.184** **(Mendelian)** **[0.3582]**
Ca_v_3.2^+/−^ × Ca_v_3.2^+/+^	**p** **=** **0.586** **(Mendelian)** **[0.0731]**	**p** **=** **0.662** **(Mendelian)** **[0.0646]**	**p** **=** **0.737** **(Mendelian)** **[0.0583]**
Ca_v_3.2^+/−^ × Ca_v_3.2^−/−^	*p* *<* *0.001* *(non-Mendelian)* *[0.9369]*	*p* *=* *0.022* *(non-Mendelian)* *[0.5239]*	*p* *=* *0.002* *(non-Mendelian)* *[0.7901]*

(B) Ca_v_2.3 Breeding scheme	♂ + ♀	♀	♂
Ca_v_2.3^+/−^ × Ca_v_2.3^+/−^	**p** **=** **0.076** **(Mendelian)** **[0.5171]**	**p** **=** **0.079** **(Mendelian)** **[0.5096]**	**p** **=** **0.628** **(Mendelian)** **[0.1265]**
Ca_v_2.3^+/−^ × Ca_v_2.3^+/+^	**p** **=** **0.560** **(Mendelian)** **[0.0766]**	**p** **=** **0.082** **(Mendelian)** **[0.3236]**	**p** **=** **0.374** **(Mendelian)** **[0.1143]**
Ca_v_2.3^+/−^ × Ca_v_2.3^−/−^	**p** **=** **0.292** **(Mendelian)** **[0.1427]**	**p** **=** **0.639** **(Mendelian)** **[0.0669]**	**p** **=** **0.304** **(Mendelian)** **[0.1379]**

No alterations were detected for the Ca_v_3.2^+/−^ × Ca_v_3.2^+/+^ scheme (with 273 offspring from 43 litters, Fig. 1B_I_, Table 1A, Suppl. Tab. 1). The same held true for the sex-specific analysis (Fig. 1B_II_ for ♂, Fig. 1B_III_ for ♀, Table 1A, Suppl. Tab. 2, 3).

For the Ca_v_3.2^+/−^ × Ca_v_3.2^−/−^ breeding (with 309 offspring from 62 litters), a deviation from the Mendelian inheritance pattern was detected for both sexes as well as in the sex-specific analysis. There turned out to be an increase of Ca_v_3.2^+/−^ and a decrease of Ca_v_3.2^−/−^ mice compared to the Mendelian distribution (Fig. 1C_I_, C_II_, C_III_, Table 1A, Suppl. Tab. 1–3). For the three different breeding schemes, a significant alteration in litter size was only observed for both sexes, but not for separate analysis of male and female offspring (Table 2A).

**Table 2:** Litter sizes in Ca_v_3.2 and Ca_v_2.3 null mutant breeding. (A) Litter sizes for Ca_v_3.2^+/−^ × Ca_v_3.2^+/−^, Ca_v_3.2^+/−^ × Ca_v_3.2^+/+^ and Ca_v_3.2^+/−^ × Ca_v_3.2^−/−^ breeding schemes. (B) Litter sizes for Ca_v_2.3^+/−^ × Ca_v_2.3^+/−^, Ca_v_2.3^+/−^ × Ca_v_2.3^+/+^ and Ca_v_2.3^+/−^ × Ca_v_2.3^−/−^ breeding schemes. All values are displayed as mean ± SEM.

(A) Ca_v_3.2 Breeding scheme	♂ + ♀	♀	♂
Ca_v_3.2^+/−^ × Ca_v_3.2^+/−^	5.93 ± 0.36	3.07 ± 0.25	3.07 ± 0.23
Ca_v_3.2^+/−^ × Ca_v_3.2^+/+^	6.35 ± 0.37	3.05 ± 0.21	3.64 ± 0.29
Ca_v_3.2^+/−^ × Ca_v_3.2^−/−^	4.98 ± 0.25	2.48 ± 0.17	2.88 ± 0.19
	**p = 0.0109**	**p = 0.0839**	**p = 0.0845**

(B) Ca_v_2.3 Breeding scheme	♂ + ♀	♀	♂
Ca_v_2.3^+/−^ × Ca_v_2.3^+/−^	6.35 ± 0.28	3.35 ± 0.16	3.30 ± 0.20
Ca_v_2.3^+/−^ × Ca_v_2.3^+/+^	7.00 ± 0.32	3.43 ± 0.24	3.79 ± 0.23
Ca_v_2.3^+/−^ × Ca_v_2.3^−/−^	6.81 ± 0.27	3.54 ± 0.18	3.44 ± 0.22
	**p** **=** **0.2791**	**p** **=** **0.7808**	**p** **=** **0.2923**

### Ca_v_2.3 breeding results and characteristics of inheritance

**Figure 2:**
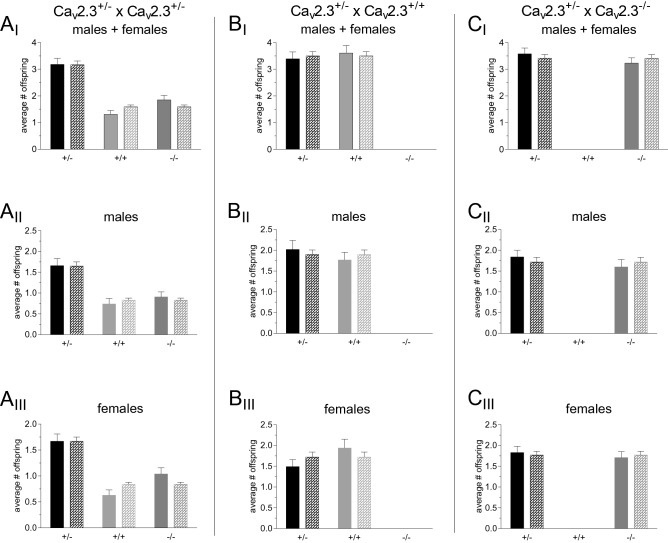
Real and theoretical average number of offspring from different Ca_v_2.3 breeding schemes. **(A**_**I**_**–C**_**I**_) Breeding results for both sexes using a Ca_v_2.3^+/−^ × Ca_v_2.3^+/−^, Ca_v_2.3^+/−^ × Ca_v_2.3^+/+^ and Ca_v_2.3^+/−^ × Ca_v_2.3^−/−^ breeding scheme. In addition, these data were analyzed separately for male offspring (**A**_**II**_**–C**_**II**_) and female offspring (**A**_**III**_**–C**_**III**_). Plain bars indicate real average offspring number, patterned bars indicate the theoretical average offspring number based on the Mendelian inheritance.

Ca_v_2.3 mutant mice were bred for about eight years in different projects of our group (see^28,31,38,39,67^). Ca_v_2.3^+/+^, Ca_v_2.3^+/−^ and Ca_v_2.3^−/−^ mice were generated using three different breeding schemes, i.e., Ca_v_2.3^+/−^ × Ca_v_2.3^+/−^, Ca_v_2.3^+/−^ × Ca_v_2.3^+/+^, and Ca_v_2.3^+/−^ × Ca_v_2.3^−/−^. In total, 1142 offspring from 170 litters were analyzed. For the Ca_v_2.3^+/−^ × Ca_v_2.3^+/−^ breeding scheme (including both sexes) with 349 offspring from 55 litters, no deviation from Mendelian inheritance was detected (Fig. 2A_I_, Table 1B, Suppl. Tab. 4). The same held true for the Ca_v_2.3^+/−^ × Ca_v_2.3^+/+^ scheme with 357 offspring from 51 litters (Fig. 2B_I_, Table 1 B, Suppl. Tab. 4). Finally, for Ca_v_2.3^+/−^ × Ca_v_2.3^−/−^ breeding (with 436 offspring from 64 litters), again no alterations from Mendelian distribution could be observed (Fig. 2C_I_, Table 1B, Suppl. Tab. 4).

Next, we carried out a sex-specific analysis of the offspring breeding results. Notably, neither in females nor in males, we observed any significant deviation from Mendelian inheritance pattern (Fig. 2A_II_, B_II_, C_II_ for ♂, Fig. 2A_III_, B_III_, C_III_ for ♀, Table 1B, Suppl. Tab. 5, 6). In summary, there is no indication of non-Mendelian inheritance in the Ca_v_2.3 mutant line described here. For the three different breeding schemes, no significant alteration in litter size was detected for both sexes and male and female offspring (Table 2B).

## Discussion

Our large-scale breeding studies for Ca_v_3.2 and Ca_v_2.3 null mutant mice have revealed a complex deviation from Mendelian inheritance for Ca_v_3.2, but no deviation from Mendelian inheritance for Ca_v_2.3 null mutants. Importantly, our results on Ca_v_3.2 mutant breeding are partially confirming previous findings from Alpdogan et al. (2020)^66^. However, our findings for Ca_v_2.3 mutant breeding are in opposite to what has been reported previously. In the following, we will discuss in detail potential reasons for the deviation from Mendelian inheritance in Ca_v_3.2^−/−^ breeding and for the discrepancies observed in Ca_v_2.3^−/−^ breeding.

### Functional implications of Ca_v_3.2 allelic loss in Ca_v_3.2 null mutant inheritance

It has recently been suggested by Alpdogan et al. (2020)^66^ that Ca_v_3.2 and Ca_v_2.3 deficient mouse lines do not exhibit Mendelian inheritance. Alpdogan et al. (2020) presented a plethora of reasons that might be responsible for this observation and the authors concluded that prenatal lethality might account for the suggested non-Mendelian inheritance. The Ca_v_3.2 mutant mouse line described by Alpdogan et al. (2020)^66^ is the same as we used in our studies. We observed a deviation from Mendelian inheritance for the Ca_v_3.2^+/−^ × Ca_v_3.2^−/−^ breeding results with a decrease of Ca_v_3.2^−/−^ mice. This observation is similar to what has been described by Alpdogan et al. (2020)^66^. In addition, a deviation from Mendelian inheritance was also detected for Ca_v_3.2^+/−^ × Ca_v_3.2^+/−^ breeding for both sexes, but not for male and female offspring separately. The latter is in contrast to what has been reported by Alpdogan et al. (2020)^66^.

In the past, breeding studies of numerous mutant mouse lines often revealed Mendelian inheritance, following Mendel’s first law, i.e., the principle of segregation, and Mendel’s second law. i.e., the principle of independent assortment^68^ (see also informatics.jax.org; https://www.komp.org). However, some mutant lines were also proven to exhibit deviation from Mendelian inheritance^69,70^. Notably, multiple reasons for exceptions to Mendelian inheritance have been characterized, e.g., polygenic inheritance, incomplete dominance, codominance, multiple alleles, pleiotrophy, epistasis, unstable/dynamic mutations, genomic imprinting, uniparental disomy, other epigenetic inheritance and gene-environment related interactions, and lethality^71–73^.

Currently, there is no scientific evidence that any of the aforementioned genetic aspects could be responsible for the exceptions to Mendelian inheritance in Ca_v_3.2 null mutant breeding. One aspect that justifies special attention is the functional involvement of Ca_v_3.2 VGCCs in sperm and oocyte physiology.

In many species including mice, molecular, pharmacological and electrophysiological studies suggested that VGCCs are involved in spermatogenesis and sperm function, particularly sperm motility and the acrosome reaction^74–84^. The mammalian acrosome reaction is Ca^2+^ dependent and requires a complex spatio-temporal activation of different entities of Ca^2+^ influx, i.e., via Ca_v_3.2 VGCCs, IP_3_ receptors, and TRPC2 channels^85,86^. Early reports suggested the presence of both Ca_v_3.1 and Ca_v_3.2 VGCCs in sperm^87^. However, the dominant T-type Ca^2+^ currents in spermatogenic cells turned out to be related to Ca_v_3.2, as Ca^2+^ current density in spermatogenic cells was not reduced in Ca_v_3.1^−/−^ mice compared to control animals^87^. Furthermore, studies in testes from immature and adult mice revealed a complex spatio-temporal transcription pattern for Ca_v_3.2 VGCCs^88^. The Ca_v_3.2 function in murine spermatogenesis, sperm motility, capacitation and acrosome reaction was not further evaluated for the potential consequences on breeding upon Ca_v_3.2 ablation^74,89,90^. However, inhibition of spermatogenic T-type Ca^2+^ channels by genistein was shown to attenuate mouse sperm motility and acrosome reaction^91^.

Importantly, the spatio-temporal fine tuning of Ca^2+^-influx is also critical in maturing oocytes and eggs and proper mammalian development post fertilization^92^. The mouse egg remains arrested at metaphase of the second meiotic division until fertilization triggers sustained Ca^2+^ oscillations^92,93^. These oscillations are critical for the activation of embryonic development in mice^93–98^. Bernhardt et al. (2015) demonstrated in mouse eggs that Ca_v_3.2 VGCCs are a prerequisite for proper accumulation of Ca^2+^ during oocyte maturation, for Ca^2+^ influx following fertilization, and for proper egg activation^92^. In Ca_v_3.2^+/+^ eggs, characteristic T-type Ca^2+^ currents were detected which are in accordance with previous studies^99^. As expected, T-type Ca^2+^ currents were reduced by 44% in Ca_v_3.2^+/−^ eggs (compared to Ca_v_3.2^+/+^ eggs) and not measurable in Ca_v_3.2^−/−^ eggs. Thus, Ca_v_3.2 VGCCs seem to represent the only functional T-type Ca^2+^ channel in mouse eggs with severe impact on Ca^2+^ homeostasis and dynamics^92^. Importantly, the Ca_v_3.2^−/−^ mouse line was originally reported to be viable and fertile^60^. Recent analysis of fertility revealed that the number of pups per litter was significantly reduced in Ca_v_3.2^−/−^ females compared to Ca_v_3.2^+/+^ females^92^. These findings are in accordance with the results of our large-scale breeding studies in which a reduced litter size from Ca_v_3.2^−/−^ females was detected. Also, the results of Bernhardt et al. (2015) are in line with our observation of fewer homozygous mutant mice than expected in the Ca_v_3.2^+/−^ × Ca_v_3.2^−/−^ breeding scheme and a relative increase in Ca_v_3.2^+/−^ mice^92^. As Ca_v_3.2 null mutant mice are not completely infertile, it was also suggested that additional Ca^2+^ entry mechanisms may act as a partial compensatory mechanism to sustain Ca^2+^ oscillations^92^.

Current scientific data point to the fact that the favorite explanation for the observed deviation from Mendelian inheritance in Ca_v_3.2 null mutant breeding originates from the important roles of Ca_v_3.2 VGCCs during oocyte maturation and following fertilization^92^ as well as the implications in spermatogenesis, sperm motility and acrosome reaction^74,77–79,89,90^. As genotyping in our study was carried out at the post weaning state, we do not have information about a potential decrease in null alleles at the pre and post-embryonic stage. Litter size analysis for our breeding schemes revealed alterations for offspring of both sexes, but not for separate analysis of male or female offspring (Table 2A). We cannot comment on knockout and wild-type litter sizes, as we did not breed Ca_v_3.2^−/−^ × Ca_v_3.2^−/−^ or Ca_v_3.2^+/+^ × Ca_v_3.2^+/+^. In summary, transmission ratio distortion with biased genotype distribution and reduced litter size often gives rise to either selective embryonic lethality (impaired embryonic development at the pre- or post-implantation state) or reduced oocyte production (dysgametogenesis)^73^. Whether prenatal lethality—as previously suggested by Alpdogan et al. (2020)^66^—accounts for the reduced number of Ca_v_3.2^−/−^ mice and reduced litter size remains to be proven in the future.

### Functional implications of Ca_v_2.3 allelic loss in Ca_v_2.3 null mutant inheritance

As regards the breeding of Ca_v_2.3 deficient mice, we were not able to confirm a deviation from Mendelian inheritance as reported by Alpdogan et al. (2020)^66^. Four Ca_v_2.3^−/−^ models have been generated so far, i.e., the “Miller Ca_v_2.3 model/Chicago”^100^, the “Tanabe Ca_v_2.3 model/Tokyo”^36^, the “Schneider Ca_v_2.3 model/Cologne”^15^ and the “Shin Ca_v_2.3 model/Seoul”^37^. The genetic engineering specificities and backgrounds of all these models were reviewed in detail before by Weiergräber et al. (2006)^26^. Importantly, the mutant Ca_v_2.3 line we used here in our study (“Miller Ca_v_2.3 model”) was different from the one used by Alpodogan et al. (2020) (“Schneider Ca_v_2.3 model”)^66^. What both lines have in common is that they represent constitutive knockout models breed into C57BL/6 J mice^26^. Thus, the observed discrepancies between both inheritance studies might be based on the genetic specificities and the underlying strategies of genetic engineering of the mutant Ca_v_2.3 model described by Alpodogan et al. (2020)^66^ and the Ca_v_2.3 null mutant model that we used. The mutant Ca_v_2.3 line which our study is based on, was the first Ca_v_2.3^−/−^ model to be described in literature^100^ and is widely used in the scientific community^31,38,39,101^. Based on the gene inactivation strategy in this model, the potential existence of a protein remnant/fragment, i.e., a truncated form of Ca_v_2.3 cannot be fully ruled out. However, there is no evidence that such truncated forms of Ca_v_2.3 are expressed and thus their existence remains speculative^100^. Importantly, it has been demonstrated that neither fragments of domain I-II or domain III-IV of, e.g., Ca_v_2.2, another HVA non L-type Ca^2+^ channel closely related to Ca_v_2.3, can form functional channels when expressed individually, together with accessory subunits such as β_1b_ and α_2δ1_^102^. Therefore, there is no molecular, biochemical or electrophysiological evidence that suggests or even proves the formation of functional Ca_v_2.3-like channels based on potential two domain fragments in the model we used. Also, there are no indications that such potential fragments could be cytotoxic and influence the inheritance pattern. Notably, we previously checked for compensatory mechanisms in the Ca_v_2.3^−/−^ model (“Miller Ca_v_2.3 model”) and carried out real-time PCRs on other VGCCs^31^. We also performed micro-array analysis of brains from our Ca_v_2.3^+/+^ and Ca_v_2.3^−/−^ animals that also did not reveal significant compensatory up- or down-regulation of other genes in the Ca_v_2.3^−/−^ model described in Wilson et al. (2000)^100^. In summary, we do not have evidence that the genetic manipulation of the Ca_v_2.3 null mutant line used here affects the inheritance pattern.

Importantly, the Ca_v_2.3^−/−^ mice described by Alpdogan et al. (2020)^66^ might also generate a protein remnant, i.e., a N-terminal Ca_v_2.3 peptide fragment^15^. The N-terminus of Ca_v_2 Ca^2+^ channels is not only involved in G-protein regulation but also responsible for dominant negative (cross-) suppression of Ca_v_2 channels in general^103^. It is essential to note that a reduction/elimination of Ca_v_2.3 expression shown by Western blotting using antibodies directed against domain I or domain IV does not rule out the potential existence of such an N-terminal protein fragment in this model (“Schneider Ca_v_2.3 model”)^15^. However, the existence of such fragments and their potential devastating impact on e.g., gametogenesis (spermatogenesis/oogenesis) remains speculative as well. Given the lack of available micro-array data from this model, compensatory mechanisms that might account for the observed deviation from Mendelian inheritance in Alpdogan et al. (2020)^66^ cannot be ruled out.

It should also be noted that the mouse model used in Alpdogan et al. (2020) had first been described by Sochivko et al. (2002) and Pereverzev et al. (2002). The latter publications originally stated that genotyping the offspring from heterozygous Ca_v_2.3^+/−^ matings exhibited a Mendelian inheritance and that the general ablation of Ca_v_2.3 was not embryonically lethal. This suggests that other parameters, e.g. backcrossing strategies or environmental factors/changes might have interfered with their results and the obvious alterations in inheritance patterns of their Ca_v_2.3 null mutant breeding. Alpdogan et al. (2020) did not further comment on this contradictory description of the inheritance pattern in their model.

Given the important physiological roles of Ca_v_2.3 R-type VGCCs, e.g., in the cardiovascular system and germ cell physiology, it is tempting to hypothesize that ablation of this channels might have severe effects on prenatal development and might thus influence the inheritance pattern. In the heart for example, Ca_v_2.3 is involved in the impulse generating and conduction system, but also the autonomic cardiac control^104^. Although a number of cardiac electrophysiological alterations have been described in Ca_v_2.3^−/−^ mice using multi-electrode arrays (MEA) and radiotelemetric electrocardiographic (ECG) recordings, there are no indications that these alterations directly impair the lifespan of Ca_v_2.3 deficient mice or cause prenatal lethality^18–20,105^.

Another aspect that warrants attention is the expression of Ca_v_2.3 VGCCs in sperms. Several publications have suggested the expression of Ca_v_2.3 in mature sperms, pachytene spermatocytes and other spermatogenic cells^106,107^. In the Ca_v_2.3 null mutant model generated by Tanabe’s group, ablation of the Ca_v_2.3 Ca^2+^ channel resulted in reduced Ca^2+^ transients in the sperm head region and impaired sperm motility^107,108^. These findings also suggest that Ca_v_2.3 VGCCs contribute to the control of flagellar movement, particularly the asymmetry in flagellar beat and randomized swimming patterns^108^. The latter seems to be based on Ca_v_2.3 expression on the proximal segment of the principal piece of mouse sperm and is thus important for chemotaxic orientation^108,109^. Importantly, it turned out that the effect of Ca_v_2.3 ablation on flagellar movement was medium-dependent, e.g., on the bicarbonate concentration. Furthermore, the motility of sperms is known to depend on the complex intravaginal/intrauterine environment^110^. We are still lacking information how Ca_v_2.3^−/−^ sperm act in the in vivo environment of the mouse female reproductive tract. Notably, there might be differences in this reproductive environment between the various Ca_v_2.3 null mutant lines that affects breeding results. Interestingly, Sakata et al. (2002) reported that Ca^2+^ transient induced by KCl mediated depolarization tended to be higher in Ca_v_2.3^−/−^ sperm compared to Ca_v_2.3^+/+^ sperm. This and further findings indicate that other VGCCs might (over)compensate the lack of Ca^2+^ influx in Ca_v_2.3 null mutant sperms^108,111–113^. Sakata et al. (2002) also did not report about an exception from Mendelian inheritance^108^. Later, Cohen et al. (2014) elaborated in detail the relevance of Ca_v_2.3 in acrosome reaction and the authors reported subfertility (smaller offspring size), e.g., in knockout breeding compared to wild-type breeding^101^. As we never bred homozygous Ca_v_2.3 null mutant mice (Ca_v_2.3^−/−^ × Ca_v_2.3^−/−^) or wild-type animals (Ca_v_2.3^+/+^ × Ca_v_2.3^+/+^), we cannot comment on these findings based on our own large-scale breeding. For our breeding schemes, analysis of litter sizes did not reveal any significant alterations, neither for offspring of both sexes, nor for male and female offspring separately (Table 2B).

It is essential to note that previous phenotyping studies on Ca_v_2.3 null mutant mice did not always reveal consistent findings. Whereas impairment of glucose tolerance and insulin release, for example, was described consistently in both the “Tanabe Ca_v_2.3 model”^114^ and the “Schneider Ca_v_2.3 model”^15^, substantial discrepancies were found for thalamocortical oscillations between the “Shin Ca_v_2.3 model”^44^ and the “Schneider Ca_v_2.3 model”^45^. The same held true for sleep architecture and circadian rhythmicity between the “Schneider Ca_v_2.3 model”^32^ and the “Miller Ca_v_2.3 model”^31^. Differences between the models might thus also affect the reproductive system.

## Conclusions

1. Our results from large-scale breeding studies partially confirm a previous report about a deviation from Mendelian inheritance in the Ca_v_3.2 null mutant line^66^. Whether this phenomenon is related to prenatal lethality—as suggested by Alpdogan et al. (2020)—cannot be specified here, as no scientific evidence is yet available to prove this hypothesis. It might be speculated that the described role of Ca_v_3.2 VGCCs in spermatogenesis, oogenesis, fertilization and embryonic development is responsible for the observed exceptions to Mendelian inheritance.

2. We cannot confirm a deviation from Mendelian inheritance in Ca_v_2.3 null mutant breeding. This discrepancy might be due to the specificities in genetic engineering in both models and related physiological consequences. Although Ca_v_2.3 VGCCs are involved in sperm physiology as well, there is no direct scientific evidence that a lack of Ca_v_2.3 alters classic inheritance. Importantly, we have no indication of prenatal lethality in the Ca_v_2.3 null mutant line that we used in our study.

3. Four different Ca_v_2.3 null mutant lines have been generated and there are examples of physiological discrepancies between these models, e.g. in the field of sleep architecture and circadian rhythmicity or in inheritance patterns as outlined in this study. Intrinsic phenomena related to the specificities of genetic engineering and compensatory mechanisms upon gene inactivation might account for such phenotypic variation. Though resource-intensive, our results suggest that physiological studies should be carried out and confirmed in more than one null mutant line if possible.

## Methods

### Ca_v_3.2 mutant mouse line

Controls (Ca_v_3.2^+/+^), heterozygous (Ca_v_3.2^+/−^) and Ca_v_3.2 deficient (Ca_v_3.2^−/−^) mice were generated from cryopreserved heterozygous embryos obtained via the Mutant Mouse Resource & Research Centers (MMRRC, supported by NIH). For further details, see MMRCC stock number 9979, strain name: B6.129-Cacna1htm1Kcam/Mmmh, strain of origin: C57BL/6 × 129, strain genetic background: C57BL/6^60,62,63^. The Ca_v_3.2 mutant mice were used in different projects of our group for several years^62–65^.

Professional breeding under state-of-the-art conditions was carried out in the central animal facility of the Federal Institute for Drugs and Medical Devices (Bundesinstitut für Arzneimittel und Medizinprodukte, BfArM, Bonn, Germany) under the aegis of the German Center for Neurodegenerative Diseases (Deutsches Zentrum für Neurodegenerative Erkrankungen, DZNE, Bonn, Germany).

All animal procedures were performed according to the guidelines of the German Council on Animal Care, and all protocols were approved by the local institutional and national committee on animal care (State Agency for Nature, Environment and Consumer Protection; Landesanstalt für Natur, Umwelt und Verbraucherschutz, LANUV, Germany, AZ 87-51.04.2010.A321, AZ 84-02.04.2013.A426). The authors further certify that all animal experimentation was performed in accordance with the National Institute of Health Guide for the Care and Use of Laboratory Animals (NIH Publications No. 80-23) revised 1996 or the UK Animals (Scientific Procedures) Act 1986 and associated guidelines, or the European Communities Council Directive of 24^th^ November 1986 (86/609/ EEC) and September 22^nd^, 2010 (2010/63/EU). In all related projects^62–65,67^, specific effort was made to minimize the number of animals used and their suffering (3R strategy).

### Breeding of Ca_v_3.2^+/+^, Ca_v_3.2^+/−^ and Ca_v_3.2^−/−^ mice

For breeding, three different approaches were performed, i.e., mating heterozygous mice (Ca_v_3.2^+/−^ × Ca_v_3.2^+/−^), heterozygous with control mice (Ca_v_3.2^+/−^ × Ca_v_3.2^+/+^) and heterozygous with knockout mice (Ca_v_3.2^+/−^ × Ca_v_3.2^−/−^). For quantitative aspects, see the “Results” section.

### Genotyping of Ca_v_3.2 mutant mice

Ca_v_3.2 mutant mice were genotyped by polymerase chain reaction (PCR) based on the protocol of the KAPA Mouse genotyping kit (Sigma Aldrich, Germany). As described previously, the following primers were used: WT-forward: 5′-ATT CAA GGG CTT CCA CAG GGT A-3′, WT-reverse/ KO-reverse: 5′-CAT CTC AGG GCC TCT GGA CCA C-3′, KO-forward: 5′-GCT AAA GCG CAT GCT CCA GAC TG -3′ (see^60,62,63^). PCRs were carried out using a C1000 thermal cycler (BioRad, Germany) with initial denaturation (94 °C for 3 min), followed by 35 cycles (denaturation, 94 °C for 15 s; annealing, 61 °C for 15 s; extension 72 °C for 15 s) and final extension (72 °C for 1 min). Finally, PCR products were separated using agarose gel electrophoresis and visualized by ChemiDoc Touch (BioRad, Germany). Examples of our genotyping of Ca_v_3.2 mutant mice are provided in detail in^62,63^. Note that genotyping of all experimental animals was carried out twice per animal (see supplementary tables 1–3) at the post weaning state. Further molecular details on the mutant Ca_v_3.2 line are also described by Chen et al. (2003)^60^. The reduction/absence of the Ca_v_3.2 expression in Ca_v_3.2^+/−^ and Ca_v_3.2^−/−^ mice was further proven by our group using the Western blot approach^62,63^.

### Ca_v_2.3 mutant mouse line

Ca_v_2.3^+/−^ embryos (kindly provided by Richard J. Miller; Department of Neurobiology Pharmacology, and Physiology; The University of Chicago; Chicago) were re‐derived with C57BL/6 J mice and maintained with random intra‐strain mating obtaining all genotypes, i.e., Ca_v_2.3^+/+^, Ca_v_2.3^+/−^ and Ca_v_2.3^−/−^ (Wilson et al., 2000). The mutant line was originally generated by the use of homologous recombination. The S4–S6 region of domain II was replaced with a neomycin/URA3 selection cassette. A null allele of Cacna1e was obtained by removal of the pore‐lining and its neighboring transmembrane regions. No Ca_v_2.3 transcript was detected in Northern blot analysis^100^ and no Ca_v_2.3 protein was found in Western blot analysis in Ca_v_2.3 knockouts^100^. The resultant Ca_v_2.3^−/−^ mice represent a constitutive knockout. The Ca_v_2.3 mutant mice were used in different projects of our group for several years^31,38,39,67^.

As for the Ca_v_3.2 mutant line, professional breeding of Ca_v_2.3 mutant mice was carried out under state-of-the-art conditions in the central animal facility of the Federal Institute for Drugs and Medical Devices (Bundesinstitut für Arzneimittel und Medizinprodukte, BfArM, Bonn, Germany) under the aegis of the German Center for Neurodegenerative Diseases (Deutsches Zentrum für Neurodegenerative Erkrankungen, DZNE, Bonn, Germany).

All animal experimentation was carried out according to the guidelines of the German Council on Animal Care, and all protocols were approved by the local institutional and national committee on animal care (State Agency for Nature, Environment and Consumer Protection; Landesanstalt für Natur, Umwelt und Verbraucherschutz, LANUV; AZ 87-51.04.2010.A321, AZ 84-02.04.2013.A426). The authors further certify that all animal experimentation was carried out in accordance with the National Institute of Health Guide for the Care and Use of Laboratory Animals (NIH Publications No. 80-23) revised 1996 or the UK Animals (Scientific Procedures) Act 1986 and associated guidelines, or the European Communities Council Directive of 24^th^ November 1986 (86/609/ EEC) and of 22^nd^September 2010 (2010/63/EU). Specific effort was made to minimize the number of animals used and their suffering (3R strategy).

### Breeding of Ca_v_2.3^+/+^, Ca_v_2.3^+/−^ and Ca_v_2.3^−/−^ mice

For breeding, three different approaches were carried out, i.e., mating heterozygous mice (Ca_v_2.3^+/−^ × Ca_v_2.3^+/−^), heterozygous with control mice (Ca_v_2.3^+/−^ × Ca_v_2.3^+/+^) and heterozygous with knockout mice (Ca_v_2.3^+/−^ × Ca_v_2.3^−/−^). For quantitative aspects, see the “Results” section.

### Genotyping of Ca_v_2.3 mutant mice

Ca_v_2.3 mutant mice were genotyped by PCR based on the protocol of the KAPA Mouse genotyping kit (Sigma‐Aldrich, Germany). The following primers were used: WT forward 5′‐GGC TGC TCT CCC AGT ATA CT‐3′; WT reverse/KO reverse 5′‐CAG GAA GCA TCA CTG CTT AG‐3′; KO forward 5′‐ATT GCA GTG AGC CAA GAT TGT GCC‐3′. PCR was carried out using the C1000 thermal cycler (Bio‐Rad) with an initial denaturation (94 °C for 3 min) followed by 35 cycles (each cycle containing the following steps: denaturation 94 °C for 15 s, annealing 59 °C for 15 s, extension 72 °C for 15 s) and final extension (72 °C for 1 min). Subsequently, PCR products were separated via agarose gel electrophoresis and detected by ChemiDoc Touch (Bio‐Rad). For details on the procedure and genotyping results see also^28,31,38,39^. Note that genotyping of all experimental mice was carried out twice per animal (see supplementary tables 4–6) at the post weaning state. Further molecular characterization of the model is provided by Wilson et al. (2000)^100^. The reduction/absence of the Ca_v_2.3 expression in Ca_v_2.3^+/−^ and Ca_v_2.3^−/−^ mice was further proven by our group using the Western blot approach^38,39^.

### Statistics

As widely used in genetics, Pearson’s chi-square test was used to check for Mendelian inheritance. The procedure applied here was described in detail by Montoliu et al. (2012)^115^ (see Table 1). Litter size analysis was carried out using One-Way ANOVA. Statistical analysis and graphical representations were conducted using GraphPad Prism (version 6) for Windows (Graphpad Software, Inc., USA). All data were displayed as mean ± standard error of the mean (SEM).

## Supplementary Information

Supplementary Figure 1.

Supplementary Figure 2.

Supplementary Table 1.

Supplementary Table 2.

Supplementary Table 3.

Supplementary Table 4.

Supplementary Table 5.

Supplementary Table 6.

## Data Availability

All relevant data are provided within this manuscript and the related supplementary information.

## Abbreviations

HT: Heterozygous
HVA: High voltage-activated
KO: Knock-out
LTP: Long-term potentiation
L-type: “Long-lasting” type Ca^2^^+^ channel
LVA: Low voltage-activated
PCR: Polymerase chain reaction
R-type: “Resistant” type Ca^2^^+^ channel
SEM: Standard error of the mean
T-type: “Transient” type Ca^2^^+^ channel
VGCC: Voltage-gated Ca^2^^+^ channel
WT: Wild-type
