# Supplementary material for: Breeding of Cav2.3 deficient mice reveals Mendelian inheritance in contrast to complex inheritance in Cav3.2 null mutant breeding
Source: Sci Rep. 2021 Jul 7;11:13972. doi: 10.1038/s41598-021-93391-6 (PMC8263769; doi:10.1038/s41598-021-93391-6)

Supplementary figure 1 (Papazoglou et al., 2021)

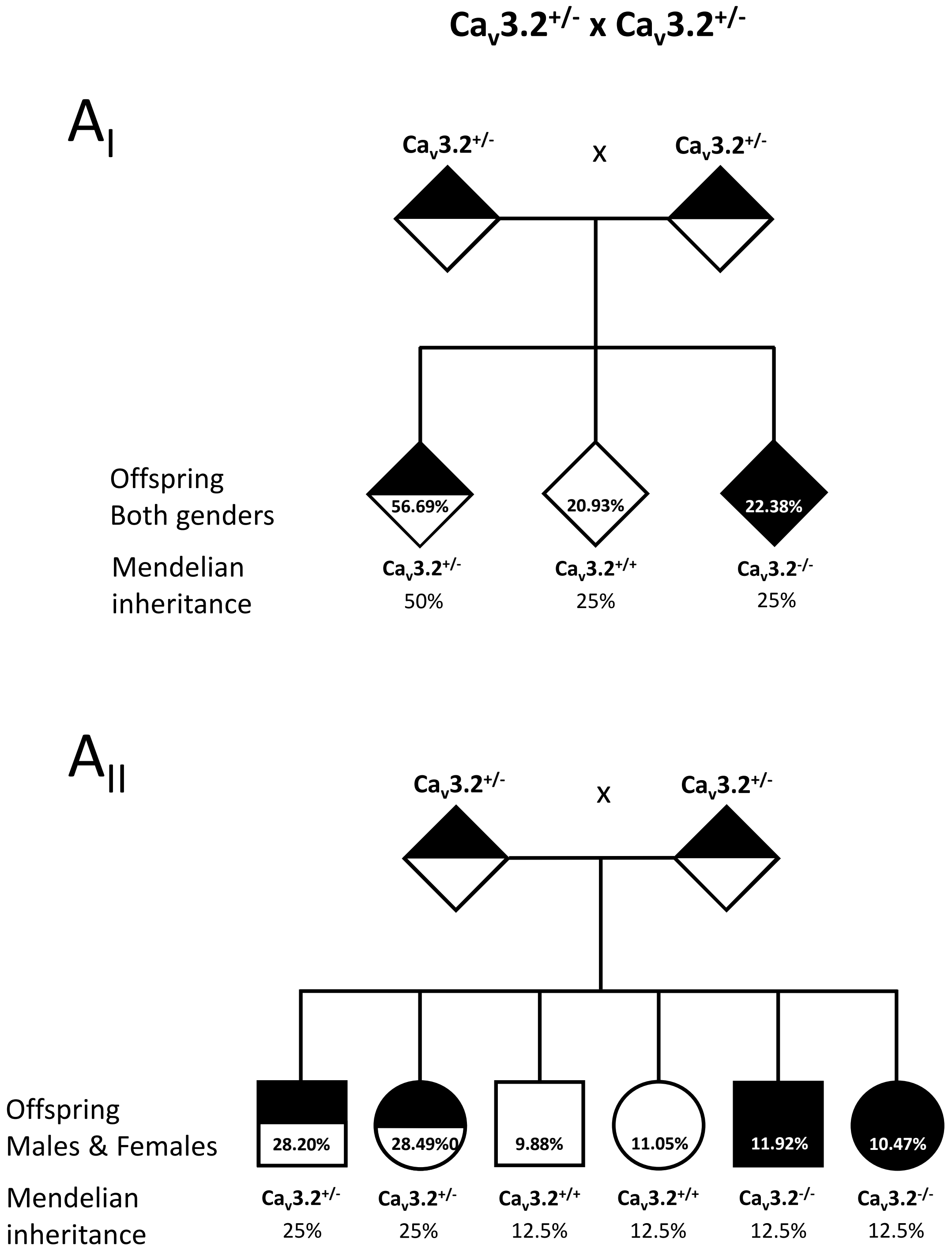

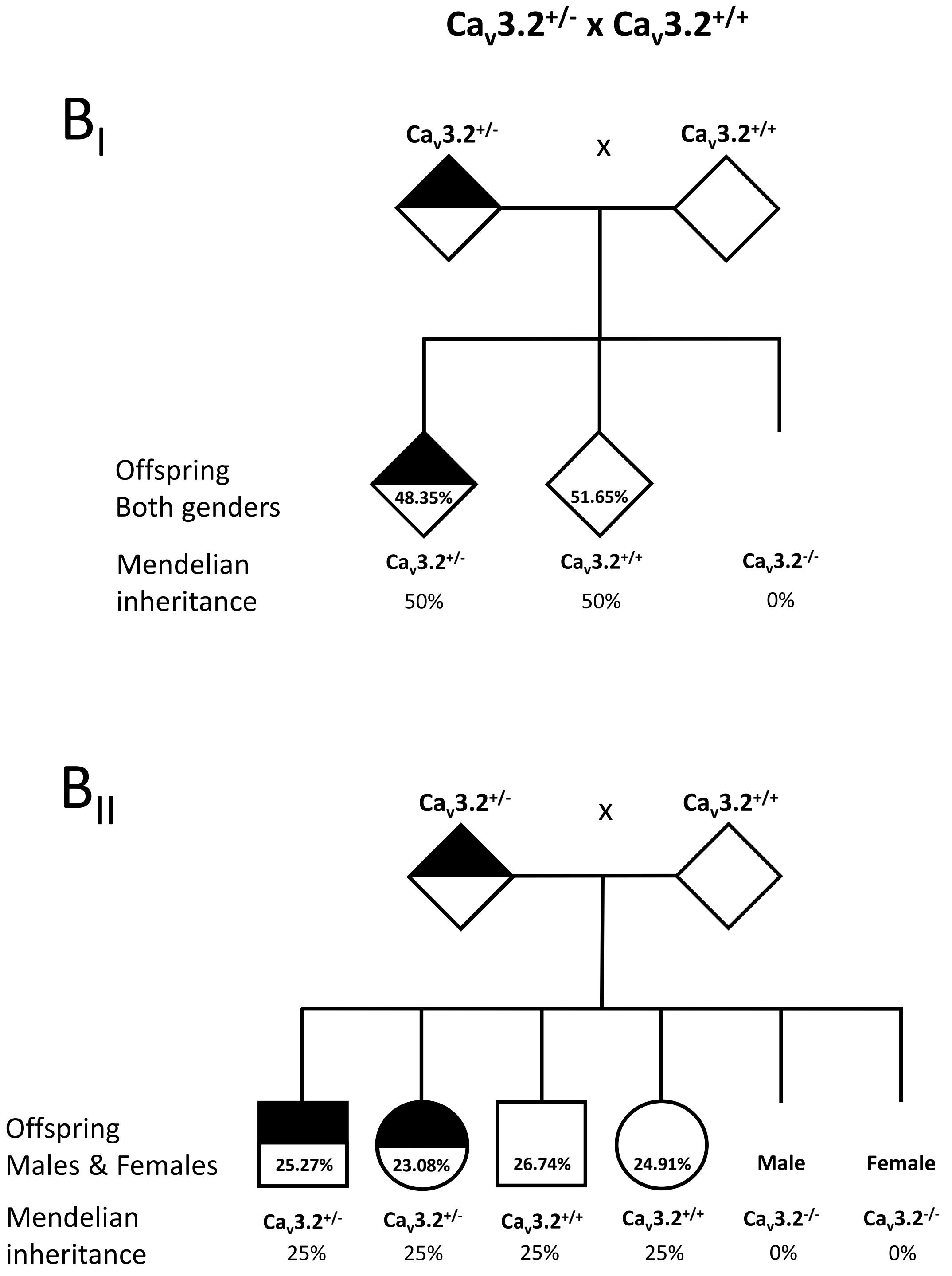
Cav3.2+/- x Cav3.2+/+
BI
Cav3.2+/-
x
Cav3.2+/+
Offspring
Both genders
48.35%
51.65%
Mendelian
inheritance
Cav3.2+/-
50%
Cav3.2+/+
50%
Cav3.2-/-
0%
BII
Cav3.2+/-
x
Cav3.2+/+
Offspring
Males & Females
25.27%
23.08%
26.74%
24.91%
Male
Female
Mendelian
inheritance
Cav3.2+/-
25%
Cav3.2+/-
25%
Cav3.2+/+
25%
Cav3.2+/+
25%
Cav3.2-/-
0%
Cav3.2-/-
0%

Supplementary figure 1 (Papazoglou et al., 2021)

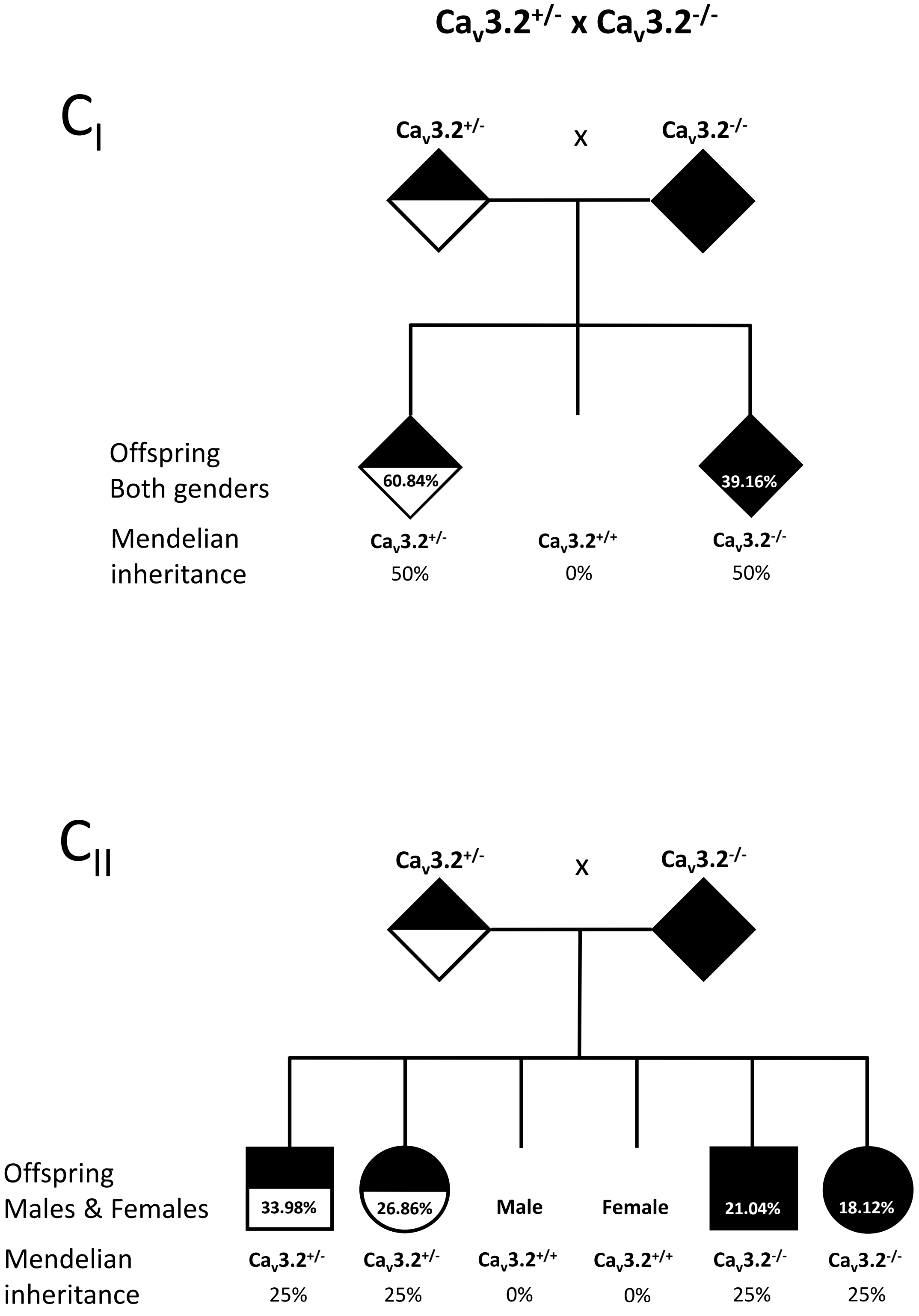

Supplement: Supplementary file 1 — Supplementary Figure 1. [file 41598_2021_93391_MOESM1_ESM.pdf]
