# Supplementary material for: Breeding of Cav2.3 deficient mice reveals Mendelian inheritance in contrast to complex inheritance in Cav3.2 null mutant breeding
Source: Sci Rep. 2021 Jul 7;11:13972. doi: 10.1038/s41598-021-93391-6 (PMC8263769; doi:10.1038/s41598-021-93391-6)

Supplementary figure 2 (Papazoglou et al., 2021)

$Ca_v2.3^{+/-}$ x $Ca_v2.3^{+/-}$

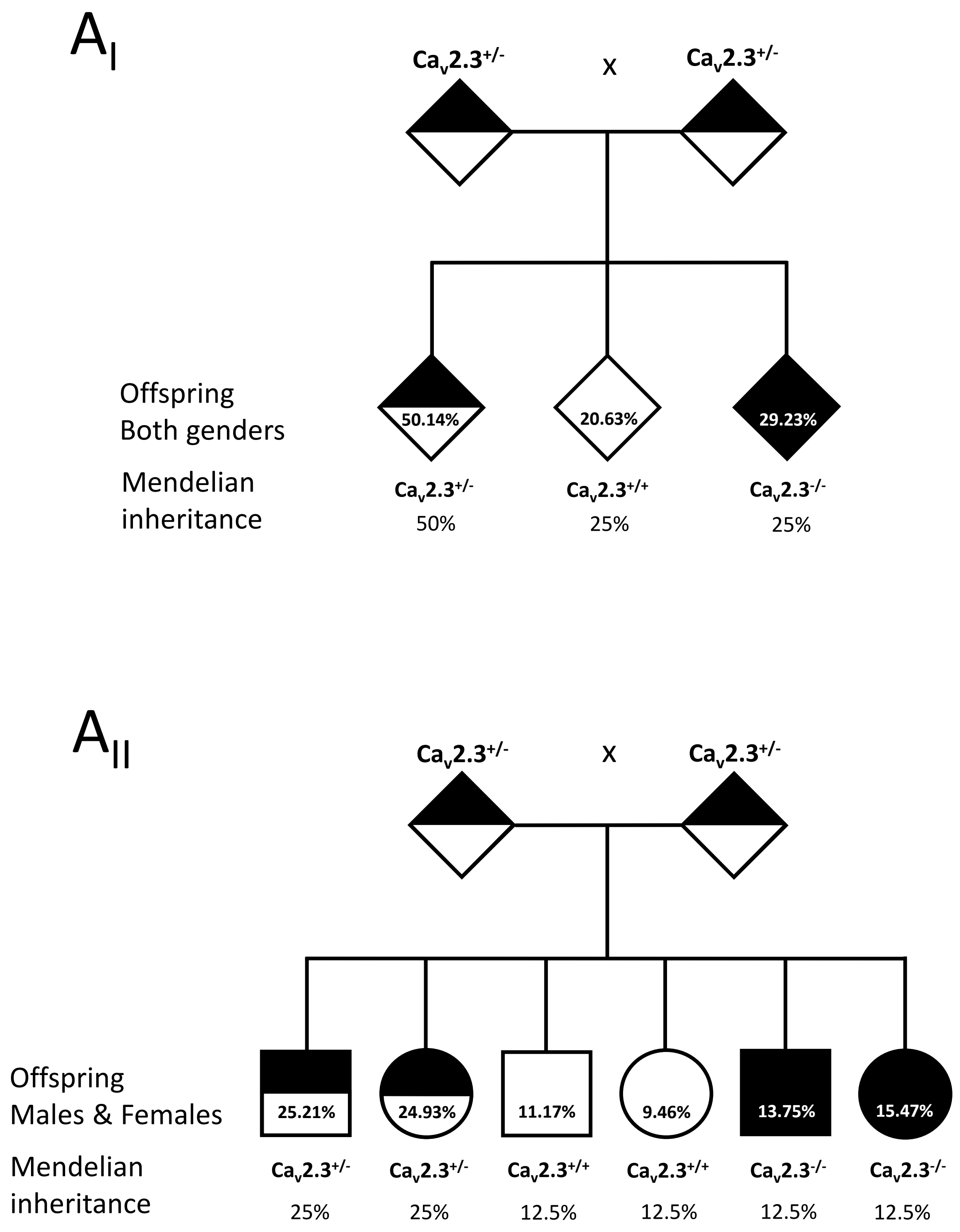

Supplementary figure 2 (Papazoglou et al., 2021)

$Ca_v2.3^{+/-}$ x $Ca_v2.3^{+/+}$

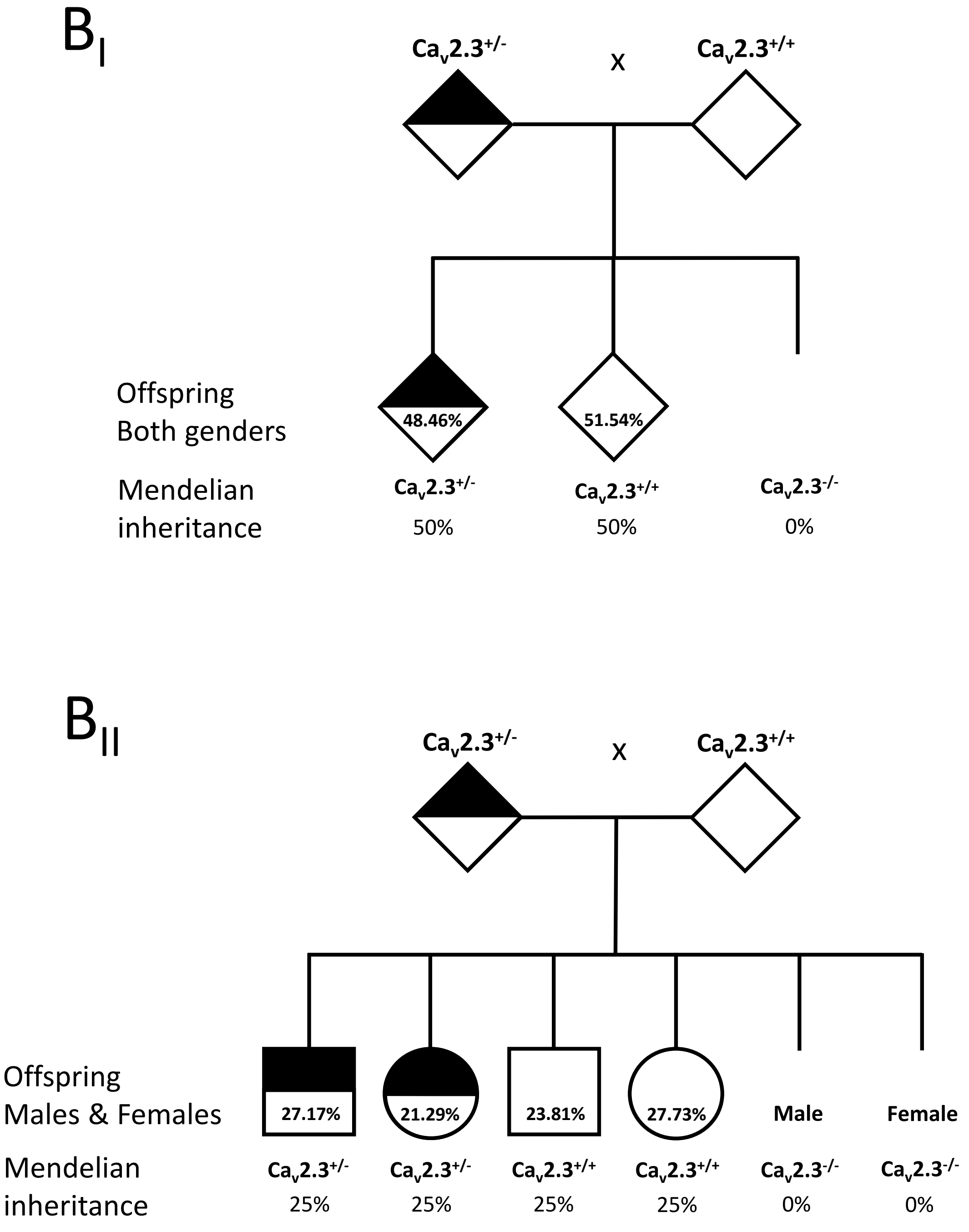

Supplementary figure 2 (Papazoglou et al., 2021)

$Ca_v2.3^{+/-}$ x $Ca_v2.3^{-/-}$

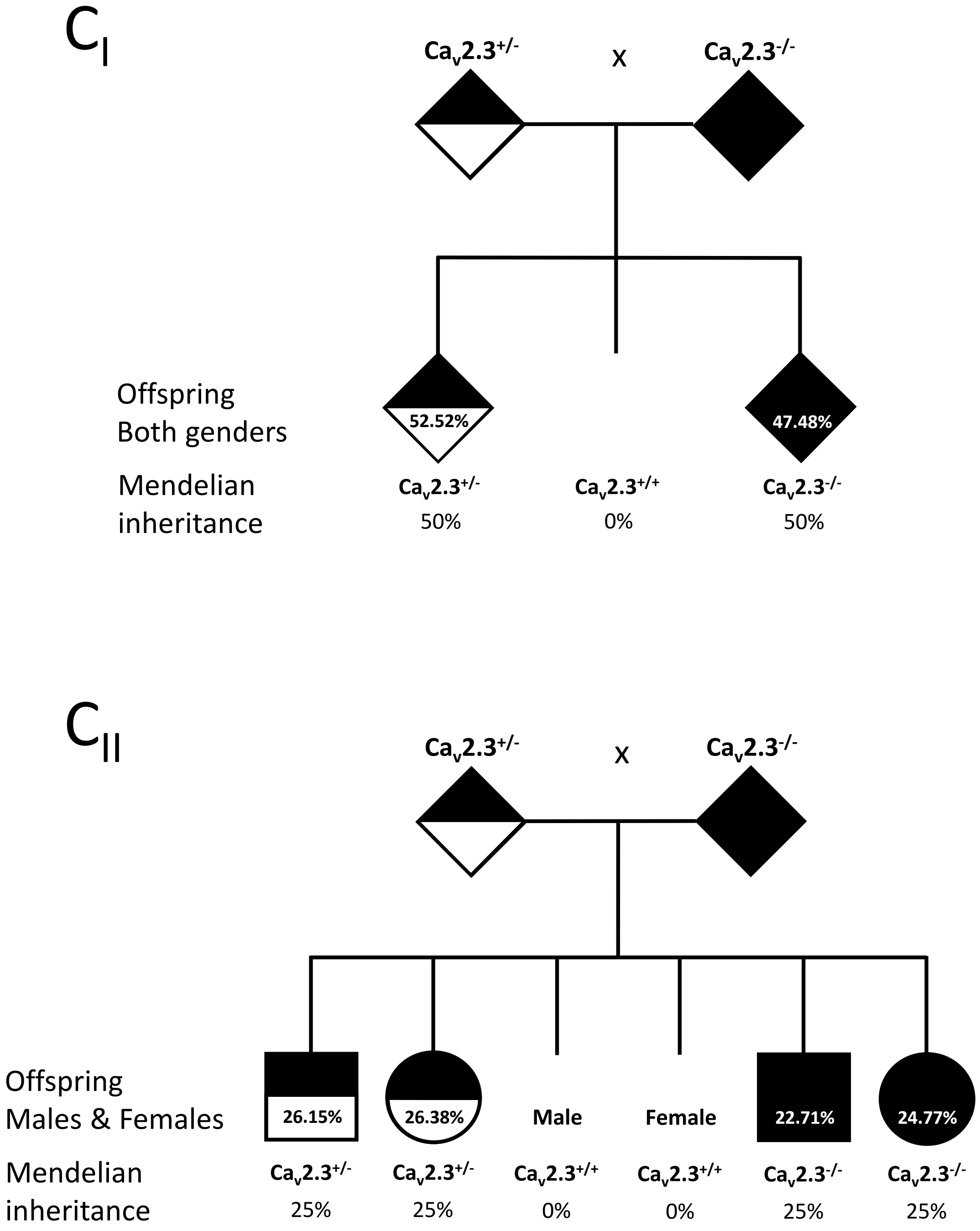

Supplement: Supplementary file 2 — Supplementary Figure 2. [file 41598_2021_93391_MOESM2_ESM.pdf]
